# Neuralgia

**Published:** 1872-03

**Authors:** N. E. Wallace


### NEURALGIA.

BY N. E. WALLACE, M. D.

In nine cases out of ten of supposed neuralgia, the extraction of some badly decayed tooth, which the suffering individual knows ought to have been extracted more than a year ago, perhaps, would cause a subsidence of all symptoms of neuralgia. As a prominent example of the above, I am induced to report the following case: Mrs. M. B. a widow, resident of this city (Boston), of very delicate constitution, was attacked with severe pain in the right side of the head, neck and shoulder, about twenty months ago, and from the severity of the pain and other circumstances attending it, she came to the conclusion that it was neuralgia, and by consulting with her medical adviser, her opinion was confirmed. She used, therefore, all possible remedies for that disease without success. In the meantime her attacks were growing more frequent, and more severe; and for the last two or three months they occurred daily at precisely three o'clock in the afternoon, and continued with the most intense severity until about nine o'clock, when the pain would begin gradually to subside, growing less and less until she was perfectly easy. At this stage of the disease she was in Augusta, Me., whether in search of medical advice or not, I do not know, but while there she consulted an eminent physician of that city, who advised her to have her teeth examined, intimating that they might be involved. He gave her at the same time a prescription for neuralgia to be used in case the teeth were not at fault. With this advice she returned home, and called on me, and related substantially what I have stated above, I examined her teeth, and found the inferior wisdom tooth of the right side decayed to the nerve, and I gave it as my opinion that all her neuralgia originated there; I therefore, advised its immediate removal to which she assented.

The first day after the tooth was extracted, she had very little pain, the next still less, and on the fourth none at all. Thus a perfect cure was effected of what, perhaps, nineteen out of twenty of our very best physicians would have pronounced neuralgia without once thinking of the teeth.

I do not offer the above as a case of rare occurrence, I have met with several such in the course of my dental practice, as doubtless, dentists in general have, and I can not account for the fact, that physicians so generally prescribe for neuralgia without once thinking of the teeth, where there is so striking a similarity to true neuralgia in many cases of toothache.

